# Microfluidic chips for decoding cancer-immune crosstalk in immunotherapy

**DOI:** 10.3389/fimmu.2026.1825593

**Published:** 2026-05-14

**Authors:** Xianlei Cai, Xia Xiao, Congcong Zhang, Weiming Yu

**Affiliations:** Department of Gastrointestinal Surgery, The Affiliated Lihuili Hospital of Ningbo University (Ningbo Medical Center Lihuili Hospital), Ningbo, Zhejiang, China

**Keywords:** cancer-immune interaction, microfluidics, precision oncology, tumor microenvironment, tumor-on-a-chip

## Abstract

The crosstalk between cancer cells and the immune system within the tumor microenvironment (TME) governs the efficacy of immunotherapeutic interventions. However, conventional preclinical models fail to recapitulate these dynamic processes. Microphysiological systems, particularly immunocompetent tumor-on-a-chip (TOC) platforms, bridge the gap between simplified two-dimensional cultures and animal models. These devices integrate microfluidic engineering, biomimetic extracellular matrices, and controlled perfusion. These platforms effectively recapitulate the cellular heterogeneity, three-dimensional structure, and physiological flow conditions of the TME. This review examines the engineering principles of immunocompetent TOC platforms and their applications in cancer immunotherapy research. These systems enable mechanistic studies of the cancer-immunity cycle, including immune cell recruitment, migration, and tumor cell cytotoxicity. They are particularly valuable for evaluating cell-based immunotherapies, including CAR-T cells. TOC platforms also facilitate drug screening and the testing of combination therapies. They show promise for functional precision oncology when integrated with patient-derived cells. Recent advances have extended these models toward greater physiological complexity. For example, multi-organ-on-a-chip systems capture systemic interactions, while lymph node-on-a-chip platforms enable studies of immune activation, and additionally organ-specific models mimic metastatic sites. Despite their potential, challenges remain in standardization, clinical validation, and balancing complexity with experimental control. As the field addresses these limitations through collaboration and integration with advanced analytics, immunocompetent TOC platforms are poised to become essential tools for understanding cancer-immune biology and accelerating personalized immunotherapy

## 1 Introduction

The emergence of cancer immunotherapy, which uses the patient’s own immune system to fight malignancies, has significantly changed the oncology landscape. Immune checkpoint inhibitors (ICIs), chimeric antigen receptor (CAR) T cells, and other immunomodulatory agents have demonstrated remarkable outcomes in subsets of patients (1–3). However, the clinical success of these therapies remains inconsistent, with response rates varying significantly across cancer types and individual patients (2, 3). A primary obstacle is the complexity and heterogeneity of the tumor microenvironment (TME), a complex environment comprising cancer cells, stromal components, immune cells, extracellular matrix (ECM), and vasculature (4–6). The TME is not simply an inactive structure but an active participant in tumor progression, promoting immune evasion and resistance to therapy (3, 7). This complex interplay between cancer and immunity, conceptualized as the cancer-immunity cycle, involves a series of coordinated steps from antigen presentation to immune cell trafficking and effector function, each susceptible to dysregulation by the tumor (8). Traditional models often fail to capture this complexity. Two-dimensional (2D) cultures lack the three-dimensional (3D) architecture and biochemical gradients of living tissues (9–11). While animal models provide systemic context, interspecies differences limit their predictive value for human responses (7, 12, 13). Similarly, patient-derived xenografts (PDXs) are restricted by high costs and the lack of a functional human immune system (13, 14). This disconnect between preclinical results and clinical efficacy remains a critical barrier (2, 7).

This requirement has driven the development of advanced *in vitro* models that can more accurately recapitulate human physiology. Among these, microphysiological systems (MPS), particularly organ-on-a-chip (OOC) and tumor-on-a-chip (TOC) platforms, have emerged as transformative tools (6, 15, 16). By using microfluidic engineering, biomaterials, and 3D cell culture, these systems enable precise spatial control over cellular organization, dynamic perfusion mimicking blood flow, and the integration of mechanical forces (15, 17, 18). When these chips are engineered to incorporate relevant immune cell populations and become immunocompetent, they offer a new opportunity to study the interactions between cancer and the immune system with high spatial and temporal resolution (5, 15, 19). This review explores how immunocompetent TOC platforms are addressing the limitations of traditional models, their engineering principles, their diverse applications in immunotherapy research, and the future challenges and opportunities for their clinical translation.

## 2 Limitations of conventional preclinical models and the rise of microphysiological systems

The effort to understand and therapeutically manipulate the tumor-immune microenvironment has long been impeded by the existing limitations of available model systems (**Table 1**). Traditional 2D monolayer cultures, while simple and high-throughput, fail to capture the dimensionality and cell-matrix interactions critical for cellular phenotype and function. Cancer cells cultured on plastic often exhibit changed gene expression profiles, metabolism, and drug sensitivity compared to their *in vivo* counterparts (20). More critically, these models cannot replicate the structured interactions between tumor, stromal, and immune cells that define the TME and determine immunotherapy outcomes (6, 9). The absence of a vascular component and interstitial flow further limits studies on immune cell trafficking, an important step in the cancer-immunity cycle (5, 8).

**Table 1:** Comparative analysis of conventional and advanced preclinical models for cancer-immune interaction studies.

Model type	Key advantages	Major limitations
2D cell culture	Simple to use, high-throughput, and cost-effective.	Lacks 3D architecture, cell-matrix interactions, and biochemical gradients. Inaccurate drug efficacy and immune behavior.
Animal models	Provides a whole-organism, systemic context.	Significant interspecies differences in immune biology and cytokine profiles. Limited predictive value for human responses.
Patient-derived xenografts	Maintains tumor heterogeneity and human tissue architecture.	Expensive and time-consuming. Often requires immunodeficient hosts, lacking a functional human immune system.
Organoids	Better preservation of cell-cell contacts, hypoxia gradients, and genetic heterogeneity.	Static conditions lacking controlled perfusion, mechanical signals, and vascular components.
Tumor-on-a-chip	Precise spatial control of the TME, dynamic perfusion, and integration of mechanical forces. Enables real-time visualization of immune cell recruitment and killing.	Technical complexity in device fabrication and multi-cell co-culture. Current lack of standardization and clinical validation.

Animal models, especially immunocompetent mice, provide a whole-organism context but introduce species-specific differences. Mouse immune systems differ from humans in lymphocyte subsets, cytokine profiles, and checkpoint molecule expression, complicating the application of immunotherapy responses (12, 21). While humanized mouse models implant human immune cells, they are complex, variable, and do not fully reconstruct human tissue architecture and physiology (13). PDXs maintain tumor heterogeneity but are costly, have low implantation rates, and typically require immunodeficient hosts, removing the possibility of studying functional human immune responses (14). Similarly, patient-derived cancer cells (PDCs) in culture lose stromal and immune components, along with spatial organization (14).

The acknowledgement of these limitations has promoted innovation towards 3D culture systems, such as spheroids and organoids (3, 6). Tumor spheroids and patient-derived organoids (PDOs) better preserve cell-cell contacts, hypoxic gradients, and some aspects of drug resistance (3, 22). Organoids, in particular, derived from patient tissue stem cells, keep genetic and phenotypic heterogeneity and have shown value in drug screening and personalized medicine (14, 22). However, even these advanced 3D models often exist in static conditions, lacking controlled perfusion, mechanical signals, and easy integration with other tissue types, such as functional vasculature or lymphoid structures (10, 14).

This is where MPS or organ-on-a-chip technology offers a fundamental change. By using microfabrication to create micron-scale channels and chambers, these platforms can contain living cells in a controlled, dynamic microenvironment (15, 16). The core advantage lies in their ability to incorporate continuous perfusion, which not only delivers nutrients and oxygen but also introduces physiological shear stress and enables the study of circulating immune cell behavior (10, 17). Furthermore, OOC platforms allow precise spatial patterning of different cell types. Endothelial cells can be arranged to form vessel-like structures, while fibroblasts and cancer cells are placed within ECM hydrogels. Immune cells can then be introduced through perfusion. Together, these components form a simplified but physiologically relevant “tumor unit” (4, 23, 24). This engineered approach provides a simplified and powerful system to separate and examine specific variables within the TME, such as the role of interstitial fluid pressure, chemokine gradients, or the effect of a specific stromal cell type on immune cell function (4, 25, 26). The development of MPS represents a coordinated effort to bridge the critical gap between simplistic *in vitro* assays and complex, low-throughput *in vivo* models, offering a human-relevant, customizable, and scalable platform for cancer immunology research (2, 16, 27).

## 3 Engineering principles and key components of immunocompetent tumor-on-a-chip platforms

The development of a physiologically relevant immunocompetent tumor-on-a-chip (ITOC) platform requires the integration of several key engineering and biological principles. The goal is to create a minimal functional unit that captures the essential features of the tumor-immune microenvironment for hypothesis-driven research (19, 28).

The foundation is the microfluidic device itself, typically made from polydimethylsiloxane (PDMS) or other polymers, and contains networks of microchannels. A common design involves adjacent channels separated by a porous membrane or, more advanced, a gel region that allows cellular and molecular crosstalk (15, 29). For instance, a central gel channel may contain the 3D tumor-stroma construct, surrounded by perfusion channels lined with endothelial cells to mimic the vasculature (17, 25). This architecture enables the independent control of the stromal and vascular compartments while allowing immune cells, introduced via the vascular channel, to interact with the tumor under flow (12, 17).

The ECM within the gel region is a critical factor of model accuracy. Natural hydrogels such as collagen I or Matrigel are widely used to provide a 3D scaffold that supports cell adhesion, migration, and signaling (10, 20). The biochemical and biophysical properties of the ECM play a key role in tumor progression and treatment response. These properties include stiffness, ligand density, and degradability. They influence cancer cell invasion, immune cell movement, and how tumors respond to therapy (1, 28). Engineered or synthetic biomaterials with adjustable properties are increasingly used to separate these variables and create defined microenvironments (28, 30).

Cellular components are selected to model specific interactions. The core typically includes patient-derived or established cancer cell lines, often pre-formed into spheroids or organoids to mimic tumor nodules (17, 31, 32). Stromal cells, such as cancer-associated fibroblasts (CAFs), are co-cultured to recapitulate the desmoplastic reaction and its immunosuppressive effects (25, 33). Advanced TOC models typically include a vascular component. This consists of an endothelial cell layer lining a perfusion channel (12, 17). This “vessel-on-a-chip” serves two important functions. It provides a physiological barrier that allows researchers to study how immune cells exit the bloodstream. It also enables the application of interstitial flow, which plays a key role in distributing chemokines and guiding cell migration (12, 34). The function of this barrier can be assessed in real-time using measurements like trans-endothelial electrical resistance (TEER) (29, 35).

Finally, the immune system is integrated to achieve immune function. This can be accomplished by adding specific immune cell populations to the perfusion medium or placing them within the stromal compartment. These populations include peripheral blood mononuclear cells (PBMCs), T cells, monocytes/macrophages, or natural killer (NK) cells (4, 15, 34). The choice of cell source is important because primary human immune cells from healthy donors or patients provide the most biologically relevant information (34, 36).

The platform can then be used to study several dynamic processes. These include T cell adhesion and movement across the endothelium (29, 36), as well as migration toward the tumor (23, 37). It also allows researchers to observe cell differentiation, such as the development of monocytes into tumor-associated macrophages (TAMs) (4, 34). Ultimately, the platform enables the study of cytotoxic functions, including killing by CAR-T cells or NK cells (17, 38, 39). Advanced sensors and real-time imaging are often integrated to monitor these events, including cytokine secretion (40), cell viability, and migratory paths (9, 24). Researchers can build increasingly advanced models by carefully combining several key elements. These include microfluidic design, biomimetic ECM, relevant cell types, and dynamic perfusion. Such models go beyond static 3D cultures to better represent the dynamic and interactive nature of the cancer-immune interaction (8, 19, 41).

## 4 Applications in cancer immunotherapy research and development

**Figure 1:**
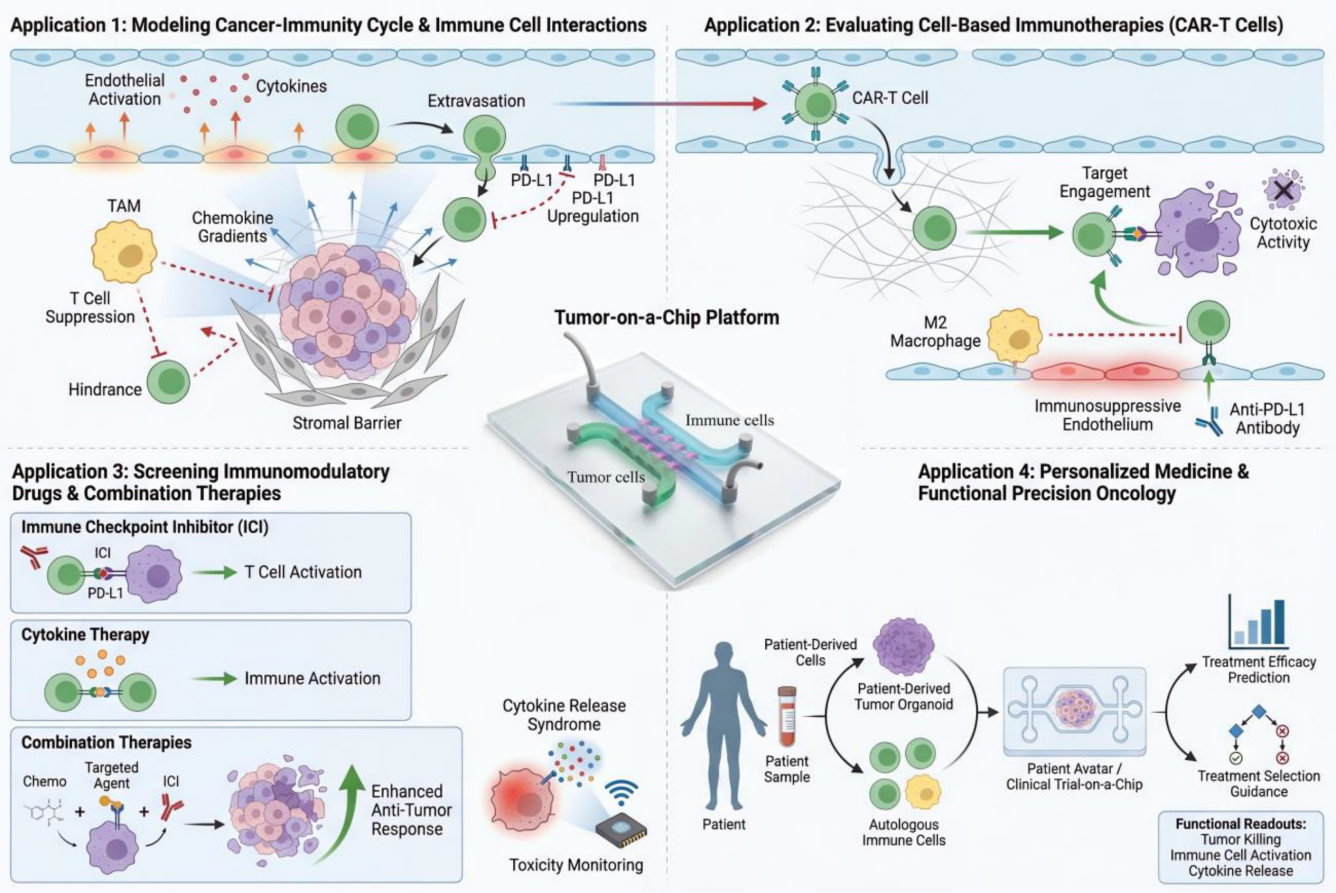
Applications of microfluidic tumor immune chips in cancer immunotherapy. Schematic overview of tumor-on-a-chip platforms for modeling tumor–immune interactions. These systems recapitulate key features of the tumor immune microenvironment, including immune cell infiltration, cytokine signaling, and immune checkpoint regulation. They enable evaluation of immunotherapies such as CAR-T cells and immune checkpoint inhibitors, support high-throughput drug screening and combination strategies, and facilitate personalized medicine using patient-derived cells to predict therapeutic responses.

Immunocompetent tumor-on-a-chip platforms have rapidly moved from proof-of-concept demonstrations to valuable tools with various applications across the immunotherapy development pipeline. Their ability to model human-specific biology with controlled complexity makes them particularly suited for mechanistic studies, therapy evaluation, and personalized medicine approaches (**Figure 1**).

### 4.1 Modeling the cancer-immunity cycle and immune cell interactions

A primary application is the analysis of the cancer-immunity cycle, the sequential process by which the immune system recognizes and destroys tumor cells (8). TOC platforms provide a high-resolution window into these complex stages by recreating essential biophysical and biochemical cues that are often absent in static cultures (8). TOC platforms allow researchers to separate and study specific phases within this cycle. For example, chips can model the extravasation of circulating lymphocytes. In such models, endothelial vessels are activated with cytokines, and the adhesion, rolling, and transmigration of perfused T cells can be visualized and quantified (29, 36). Recent studies have demonstrated that elevated interstitial fluid pressure within the tumor mass creates a physical barrier that significantly hinders this extravasation process (26). Subsequent steps, such as T cell migration through the ECM towards tumor cells guided by chemokine gradients, can also be recreated (23, 42). Furthermore, these platforms are particularly useful for examining the roles of specific immune subtypes. Studies have used TOCs to demonstrate how TAMs can promote cancer cell invasion while also suppressing T cell function, for instance by upregulating PD-L1 on adjacent endothelium (4, 12). This molecular crosstalk creates a localized checkpoint barrier at the vascular interface, effectively deactivating T cells before they encounter malignant cells (12). Similarly, models have been used to explore the complex interactions between cancer cells, fibroblasts, and immune cells. These studies reveal how stromal barriers can physically protect tumors from immune attack or secrete factors that change immune cell distribution (25). Specifically, cancer-associated fibroblasts may secrete IL-6 to promote a dense, cross-linked matrix that acts as a mechanical sieve, restricting effector cell penetration (43). This detailed understanding of cellular interactions within a human-relevant context is difficult to achieve with animal models and is essential for identifying new therapeutic targets.

### 4.2 Evaluating cell-based immunotherapies from CAR-T Cells to emerging modalities

The evaluation of adoptive cell therapies, particularly CAR-T cells, is a major focus for TOC technology. While highly effective for hematological cancers, CAR-T therapy faces significant challenges in solid tumors, including poor migration, infiltration, and survival within an immunosuppressive TME (7, 17, 39). Conventional preclinical models often fail to predict these challenges. Vascularized TOC platforms provide a unique system to study the entire CAR-T cell process. This includes vascular margination and extravasation, migration through the stroma, target engagement, and cytotoxic activity, all under physiological flow conditions (17, 38). For instance, a lung adenocarcinoma TOC was used to visualize and quantify CAR-T cell function against human tumor tissue (17). Another study on prostate cancer modeled how M2 macrophages induce an immunosuppressive endothelium that limits CAR-T cell extravasation. This effect was reversed by an anti-PD-L1 antibody (12). Beyond efficacy, TOCs are used to study resistance mechanisms and model clinical scenarios like remission, resistance, and relapse (38). They also serve as screening tools for optimizing CAR constructs, comparing different T cell sources such as those from donors versus patients, and evaluating combination strategies (38, 39).

Beyond CAR-T cells, TOC platforms are increasingly used to evaluate Natural Killer (NK) cell-based therapies (44). NK cells provide a “ready-to-use” alternative with a lower risk of cytokine release syndrome (45). However, their therapeutic potential is often limited by inefficient homing to the tumor site. Microfluidic chips allow researchers to quantify NK cell recruitment in response to chemokine gradients or tumor-modulating drugs. For example, chips have been used to study targeted migration and killing efficiency in response to CDK4/6 inhibitors in melanoma models (37). Furthermore, Tumor-Infiltrating Lymphocytes (TILs) represent a potent personalized approach for solid tumors. The primary challenge for TILs is the functional exhaustion induced by the TME (46). TOC platforms integrate patient-derived organoids and stromal cells to recreate this exhaustion *in vitro*. These models serve as high-resolution tools to test interventions designed to reinvigorate TIL function before clinical re-infusion. Looking forward, T-cell receptor (TCR) engineered T cells (47) hold significant potential for integration into TOC platforms. These systems could provide a human-relevant environment to evaluate the ability of TCR-T cells to recognize intracellular neoantigens and optimize their infiltration and persistence before clinical translation.

### 4.3 Screening and testing of immunomodulatory drugs and combination therapies

TOC platforms are becoming valuable intermediate tools for higher-throughput drug screening that surpasses 2D assays in physiological relevance. They enable the testing of immunomodulatory drugs within a structured TME. These drugs include immune checkpoint inhibitors (ICIs), cytokine therapies, and small molecule modulators (2, 16). For example, mini-tumor chip arrays containing hundreds of micro-tumors have been used to rapidly assess tumor responses to anti-PD-1 treatment. The results matched *in vivo* outcomes (31). These systems are particularly useful for evaluating combination therapies, a key element of modern oncology. Researchers can test sequences and synergies between chemotherapy, targeted agents, and immunotherapies in a controlled setting (48). A platform modeling pancreatic cancer showed that stromal-targeting with Halofuginone increased subsequent immune cell infiltration (25). Another study in an ovarian cancer TOC demonstrated that pre-treating tumors with an oncolytic virus before chemotherapy produced better outcomes compared to co-administration (48). Furthermore, TOCs can be used to evaluate the potential toxicity of immunotherapies, such as cytokine release syndrome, by monitoring pro-inflammatory cytokine profiles in real-time (40).

### 4.4 Enabling personalized medicine and functional precision oncology

Perhaps one of the most promising applications is in functional precision oncology. The integration of patient-derived cells into TOC platforms creates a “patient avatar” or “clinical trial-on-a-chip” (33, 38, 49). These cells can include tumor organoids, autologous fibroblasts, and immune cells. Such personalized models can be used to test the likely efficacy of different treatment options for an individual patient before clinical administration. A significant example is an esophagus-on-a-chip model that included patient-derived adenocarcinoma organoids and matched fibroblasts. This stroma-inclusive chip accurately predicted the patient’s response to neoadjuvant chemotherapy, performing better than static organoid cultures (33). Similarly, breast and renal cancer mini-tumor chips have been used to examine responses of primary human tumor cells to immunotherapy (31). These systems provide a functional measurement that combines the unique genetics and cellular environment of a patient’s tumor. This readout can include tumor killing, immune cell activation, and cytokine release. By offering this type of information, TOC platforms add to genomic profiling and could guide treatment selection. This is especially useful in cases where biomarkers are unclear or absent (16, 19, 49).

## 5 Advancing complexity with multi-organ chips, systemic biology, and specialized microenvironments

**Figure 2:**
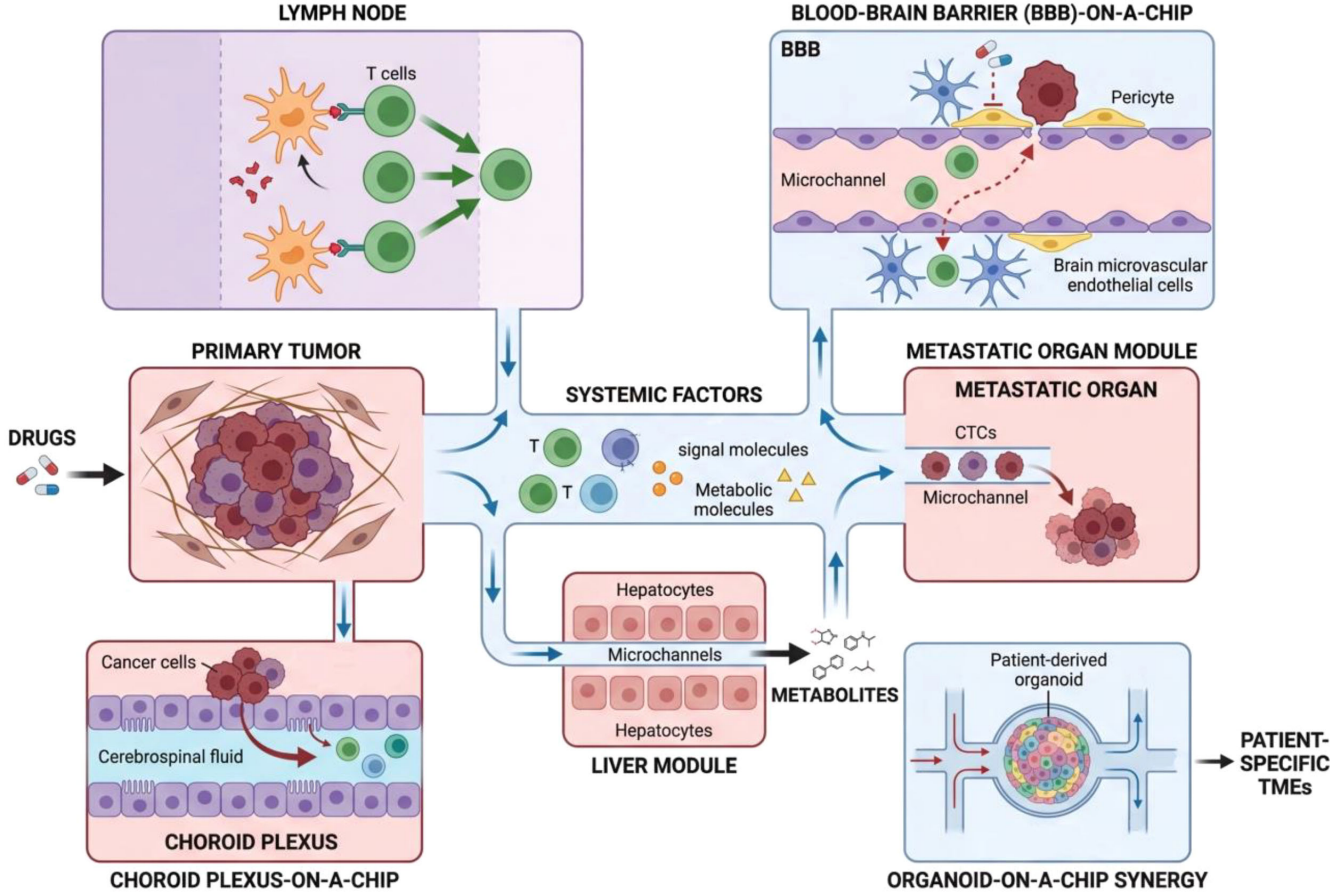
The next frontiers in tumor-on-a-chip research. This schematic illustrates the integration of multiple physiological modules to capture systemic cancer complexity. The central platform shows a multi-organ system where primary tumor and liver modules are interconnected via microfluidic flow to study the transport of systemic factors such as circulating tumor cells, T cells, and metabolic molecules. Specialized components include a lymph node-on-a-chip for studying antigen presentation and a blood-brain barrier-on-a-chip to model immune cell extravasation. A choroid plexus-on-a-chip models the interface between blood and cerebrospinal fluid to investigate metastatic seeding. The liver module utilizes hepatocytes to simulate drug metabolism and evaluate systemic metabolites. The metastatic organ module demonstrates circulating tumor cell arrest and colonization within secondary sites. Finally, the organoid-on-a-chip synergy highlights the integration of patient-derived organoids into microfluidic platforms to recapitulate patient-specific tumor microenvironments.

The next direction of tumor-on-a-chip research is moving towards increasing physiological complexity by modeling systemic interactions and specialized tissue environments (**Figure 2**). While single-organ chips are useful, cancer is a systemic disease involving metastasis and communication between distant sites. Multi-organ-on-a-chip (MOC) systems through fluid flow connect modules representing different organs. This allows for the study of pharmacokinetics, pharmacodynamics, and off-target effects in a human-relevant context (18, 50, 51). For instance, gut-liver-axis chips model first-pass metabolism and its impact on drug efficacy (50). A MOC connecting oral mucosa and skin was used to study how topical exposure to metals can cause systemic immune activation leading to skin inflammation (51). In cancer research, MOCs could model important processes like metastatic seeding. A chip connecting a primary tumor module to a “metastatic organ” module via circulating flow would allow real-time observation of circulating tumor cell arrest, extravasation, and colonization (52, 53). This is essential for understanding metastasis and testing anti-metastatic therapies.

Another key direction is the modeling of specialized immune microenvironments. The tumor microenvironment is not uniform. It differs by organ and contains unique structures. Efforts are ongoing to create chips that recreate lymph nodes. Lymph nodes are the hubs of adaptive immune activation. Lymph node-on-a-chip platforms aim to mimic several distinct structural domains. These include the cortex, the paracortex, and the medulla. Researchers can use these platforms to study antigen presentation, T cell activation, and vaccine responses (54, 55). Similarly, modeling the blood-brain barrier (BBB) and the brain TME is critical for glioblastoma research. Advanced BBB-on-a-chip models include brain microvascular endothelial cells, astrocytes, and pericytes under flow to study immune cell movement into the brain and the delivery of therapeutics (29, 56, 57). When combined with glioblastoma cells, these models can reveal how tumors impair BBB function and create an immunosuppressive environment (53, 56, 58).

Furthermore, chips are being developed to study organ-specific metastatic environments. A choroid plexus-on-a-chip was developed to mimic the specialized vasculature and cerebrospinal fluid flow of this brain region, enabling the study of breast cancer metastasis and local immune responses within this unique site (53). The combination of organoid technology with OOC is a valuable synergy, often called “organoid-on-a-chip” (59). Patient-derived organoids provide the cellular complexity and heterogeneity, while the microfluidic platform adds perfusion, mechanical signals, and simplicity of manipulation (14, 22, 60). This combination is being actively explored for diseases like lung cancer, where it can model patient-specific TMEs for drug testing (1, 59, 61).

The diversity of these models, including gut-liver, lymph node, BBB, and choroid plexus systems, shows the collective success of the broader OOC and MPS community. These platforms are not just tools for cancer research, but are part of a larger effort to map human physiology (62). In the future, the TOC field should continue to integrate other modules from the MPS community, such as bone marrow and spleen chips, to better study immune cell priming and systemic mobilization. By combining these organ modules with systemic factors like circulating hormones and metabolites, we can move from modeling localized interactions to capturing the full systemic biology of cancer (1, 27).

## 6 Current challenges, validation, and future perspectives for clinical translation

Despite their significant potential, immunocompetent tumor-on-a-chip platforms face several challenges that must be addressed to achieve their full value, particularly for clinical translation (63). A primary challenge is technical and biological complexity. Reproducibly fabricating devices, sourcing and co-culturing multiple primary human cell types, and maintaining long-term viability and functionality are difficult and can limit throughput and scalability (15, 16, 64). Currently, the field lacks unified standards for critical parameters, such as the composition and stiffness of extracellular matrix (ECM) hydrogels and the shear stress levels in vascular channels. For instance, different laboratories use varying collagen concentrations or perfusion rates, making it difficult to reproduce results across platforms (60, 64). Establishing a consensus on ‘Reference Materials’ and standardizing the source of primary human immune cells are necessary steps to ensure data reliability.

Model accuracy must be balanced with practical considerations. Increasing complexity can make models more physiologically relevant. For example, adding more cell types or mechanical forces can improve accuracy. However, this also reduces experimental control and flexibility. These changes may obscure the causal mechanisms at work (28, 41). Determining the minimal sufficient complexity for a given research question is an ongoing challenge. Furthermore, fully recreating the immune system *in vitro* is difficult. Current models typically include one or a few immune cell types. However, a fully functional immune response requires coordinated action across multiple components. These include the innate and adaptive immune systems. They also include the lymphatic system and bone marrow environments. This level of combination has not yet been achieved in any chip platform (15, 64, 65).

The most critical step towards clinical use is careful validation. To be trusted as predictive tools, TOC platforms must demonstrate a strong correlation between *in-chip* results and clinical outcomes. This requires comparison against gold-standard clinical data (2, 16). Early studies show potential. For example, a pancreatic cancer chip response to stromal-targeting therapy matched biological understanding (25), and a leukemia chip modeled clinical responses like remission and relapse (38). However, large-scale, multi-center validation studies are needed to correlate ‘in-chip’ metrics with actual patient survival rates or objective response rates. The field must develop standardized measurements and endpoints. These can include specific cytokine profiles, immune cell infiltration indices, and tumor kill ratios. Such measures must be reliably obtained across different platforms and linked to patient responses (2, 49). Benchmarking these systems against existing gold-standard animal data and clinical outcomes through blind testing will be essential to prove their predictive accuracy for personalized medicine.

Looking forward, several key directions will influence the field. Integration with advanced analytics is paramount. Combining TOCs with high-content imaging, multiplexed cytokine biosensors (40), and especially multi-omics (single-cell RNA-seq, spatial transcriptomics, metabolomics) will transform chips from observation platforms into deep discovery tools (28, 66, 67). This “omics-on-a-chip” approach can reveal molecular mechanisms behind observed phenotypes. Automation and scalability through robotics and micro-well array formats will also be important. For example, chips compatible with 96-well plates can increase throughput. This will make TOCs more practical for drug screening applications (27, 31, 68).

Cross-disciplinary collaboration between engineers, biologists, immunologists, and clinicians is essential to design clinically relevant models and interpret results meaningfully (6, 60, 61). Finally, the development of in silico models and digital twins that integrate data from TOC experiments will be valuable. Computational models can simulate complex dynamics, predict outcomes under untested conditions, and help improve chip design and experimental parameters (42, 58). As these challenges are addressed through continued innovation and collaboration, tumor-on-a-chip platforms are ready to become essential tools in the cancer immunology toolkit, accelerating the development of safer, more effective, and personalized immunotherapies.

## 7 Conclusion and outlook

The development of microfluidic organ-on-a-chip and tumor-on-a-chip platforms represents a change in cancer immunology and immunotherapy research. By transcending the limitations of conventional 2D cultures and animal models, these immunocompetent microphysiological systems offer a valuable approach to model the dynamic and spatially organized interactions within the human tumor microenvironment. From analyzing the cancer-immunity cycle and evaluating the intricate journey of CAR-T cells to screening combination therapies and enabling functional precision oncology, TOCs are demonstrating their value across the therapeutic development range (8, 19, 38).

The field is rapidly developing from modeling simple co-cultures to building advanced multi-organ systems that capture systemic biology and organ-specific metastatic environments (50, 53). The combination with patient-derived organoids, advanced biomaterials, and high-resolution analytics offers ever-greater physiological relevance and mechanistic insight (14, 22, 28). However, the path to widespread clinical use is dependent on overcoming key challenges. These include standardizing and expanding the technology, carefully validating its predictive power against clinical outcomes, and promoting deep collaboration across disciplines (2, 16, 49).

The ultimate vision is the integration of patient-specific tumor-on-a-chip models into the clinical workflow. These personalized *in vitro* models could serve as a functional diagnostic, testing a range of therapeutic options to identify the most promising regimen for an individual patient before treatment begins (33, 49). Furthermore, as tools for drug discovery, they can help reduce risk in clinical trials by providing more human-predictive data on efficacy and safety earlier in the pipeline (26, 27). While not a replacement for all preclinical models, immunocompetent tumor-on-a-chip platforms are establishing themselves as an essential complementary bridge. They offer a human-relevant, controllable, and insightful middle ground. This approach holds great promise for understanding the complexities of cancer-immune interactions and accelerating the development of the next generation of immunotherapies.
